# Supplementary material for: Trees and their seed networks: The social dynamics of urban fruit trees and implications for genetic diversity
Source: PLoS One. 2021 Mar 16;16(3):e0243017. doi: 10.1371/journal.pone.0243017 (PMC7963046; doi:10.1371/journal.pone.0243017)
Supplement: S1 Fig — Mature tree in an agroforest (above left); young tree in an orchard near Koutaba (above right). Roasted African plums and plantains sold in the streets (below left); fruit varietal diversity (below right). Pictures from the authors (1,2,4: A. Rimlinger; 3: J. Duminil). (PDF) [file pone.0243017.s001.pdf]

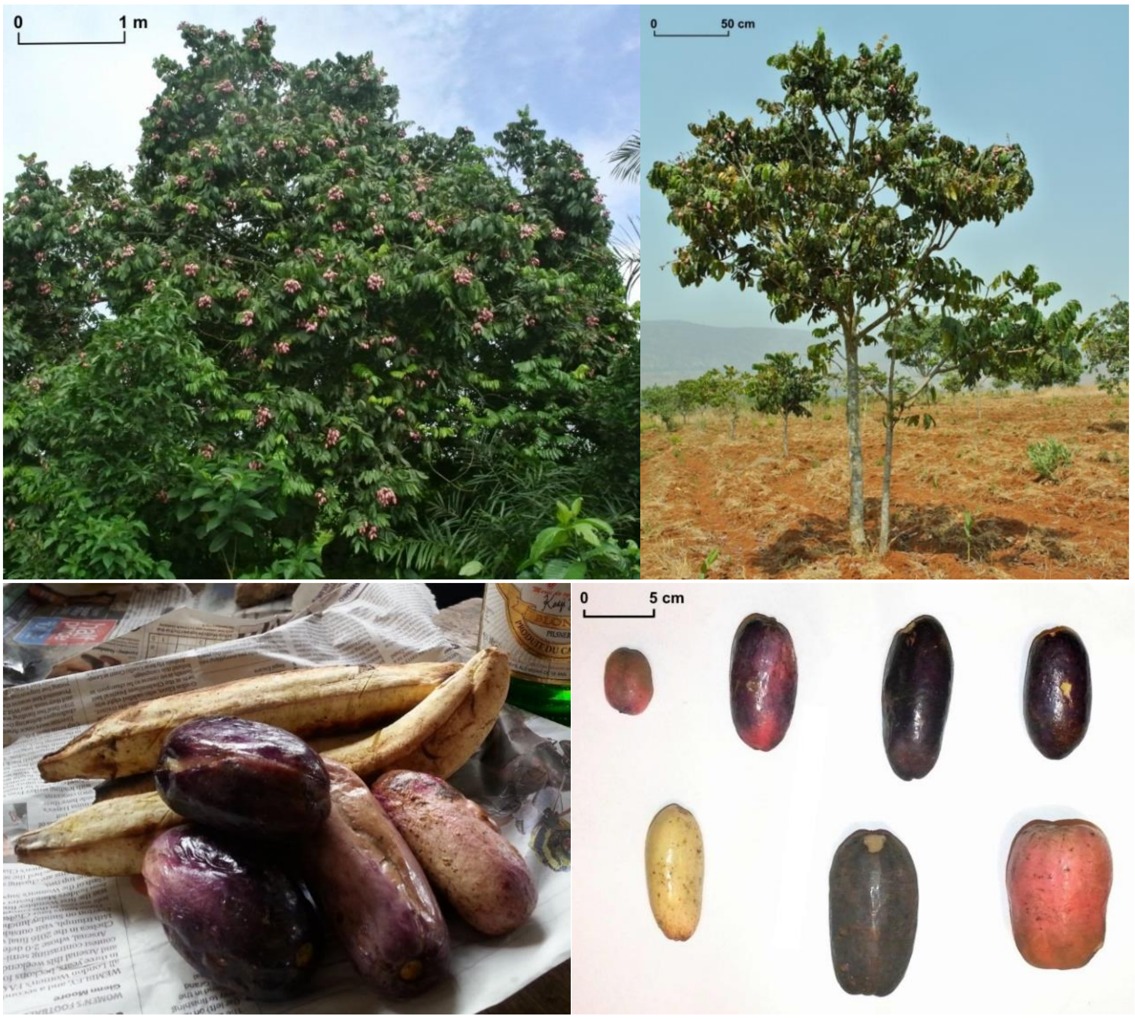

S1 Figure: Mature tree in an agroforest (above left); young tree in an orchard near Koutaba (above right). Roasted African plums and plantains sold in the streets (below left); fruit varietal diversity (below right). Pictures from the authors (1,2,4: A. Rimlinger; 3: J. Duminil).
