# Supplementary material for: Trees and their seed networks: The social dynamics of urban fruit trees and implications for genetic diversity
Source: PLoS One. 2021 Mar 16;16(3):e0243017. doi: 10.1371/journal.pone.0243017 (PMC7963046; doi:10.1371/journal.pone.0243017)
Supplement: S2 Fig — Provenance of African plums in Yaoundé main markets, accounting for more than 95% of the total volume of sold fruits reported by the sellers (1: Ndé; 2: Moungo; 3: Nkam; 4: Mbam-et-Inoubou; 5: Lékié; 6: Nyong-et-Kéllé). Dots correspond to the rural trees sampled for the genetic analysis. Administrative boundaries of Cameroon were added using a shapefile available at data.humdata.org/dataset/cameroon-administrative-boundaries. The source of the map data are stored in a database (market_provenance.txt) deposited in the OSF repository of the project. (PDF) [file pone.0243017.s002.pdf]

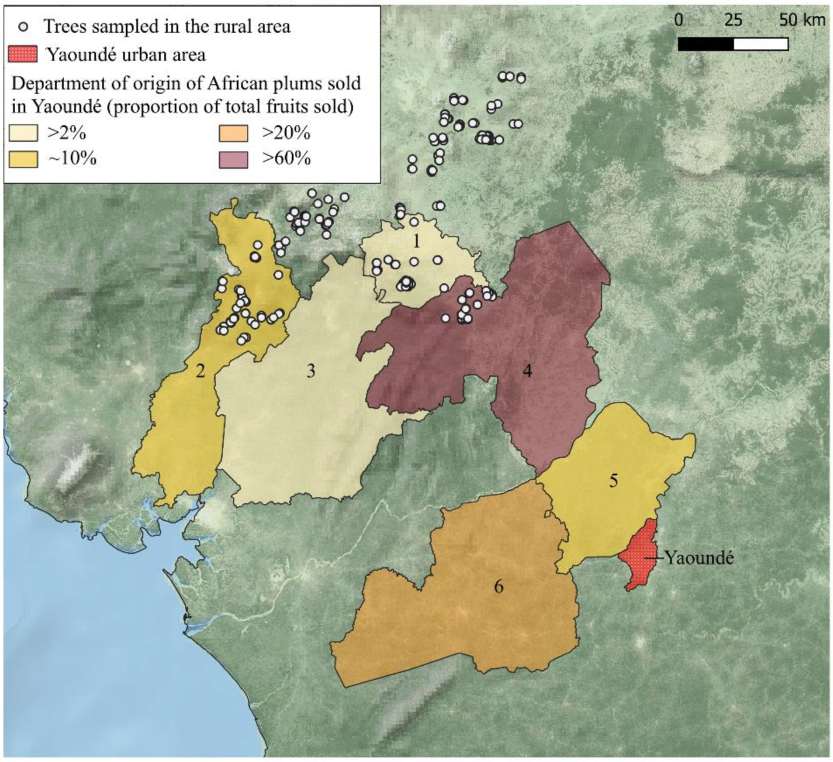


**S2 Figure**: Provenance of African plums in Yaoundé main markets, accounting for more than 95% of the total volume of sold fruits reported by the sellers (1: Ndé; 2: Moungo; 3: Nkam; 4: Mbam-et-Inoubou; 5: Lékié; 6: Nyong-et-Kéllé). Dots correspond to the rural trees sampled for the genetic analysis. Administrative boundaries of Cameroon were added using a shapefile available at data.humdata.org/dataset/cameroon-administrative-boundaries. The source of the map data are stored in a database (market_provenance.txt) deposited in the OSF repository of the project.
