# Supplementary material for: Trees and their seed networks: The social dynamics of urban fruit trees and implications for genetic diversity
Source: PLoS One. 2021 Mar 16;16(3):e0243017. doi: 10.1371/journal.pone.0243017 (PMC7963046; doi:10.1371/journal.pone.0243017)
Supplement: S3 Fig — The PCA was performed on the allele frequencies of each individual. Genotypes were clustered to show maximal differentiation along the first and second principal component (PC1 and PC2). Individuals do not cluster according to their urban or rural origin, indicating the absence of population structure. (PDF) [file pone.0243017.s003.pdf]

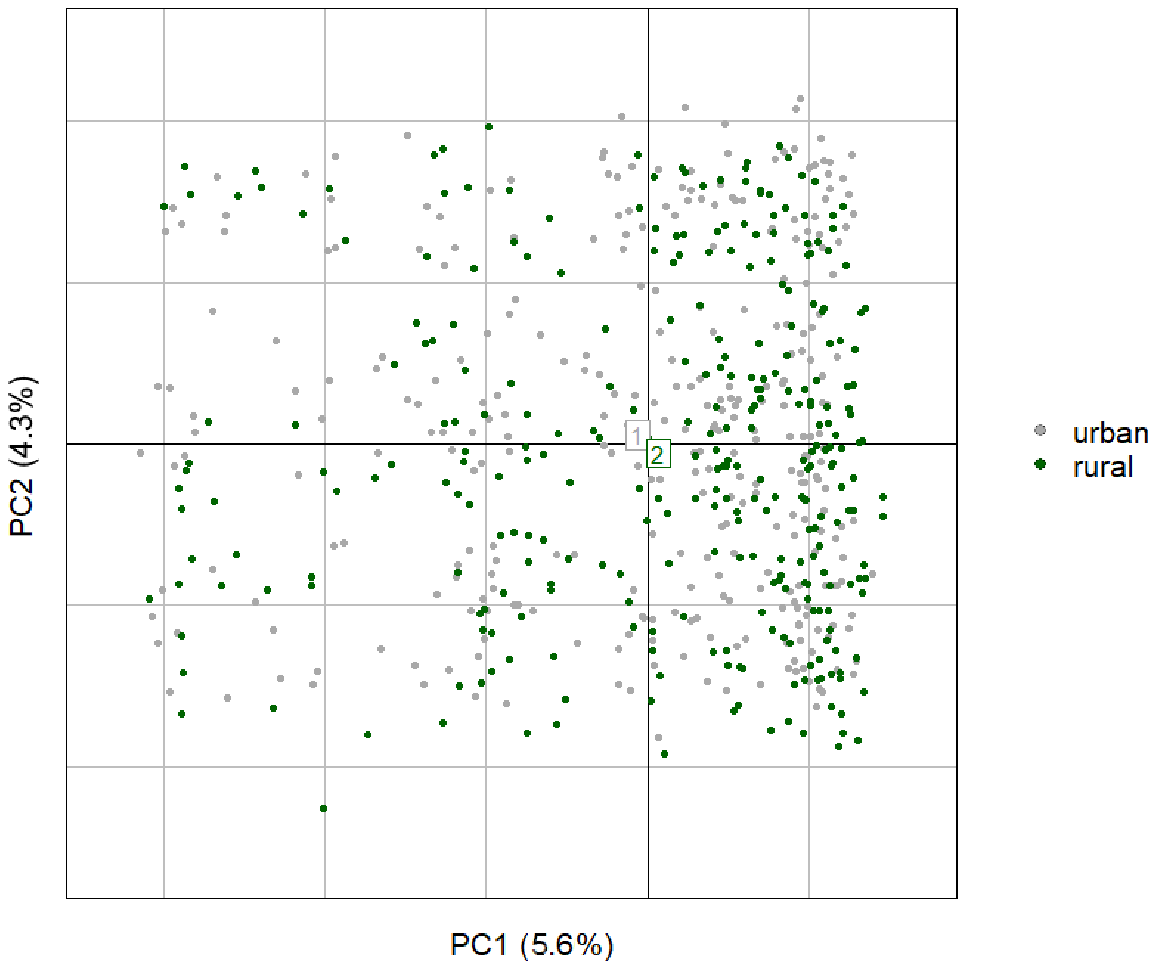

S3 Figure: Principal component analysis (PCA) of *Dacryodes edulis* microsatellite diversity from the urban and rural populations. The PCA was performed on the allele frequencies of each individual. Genotypes were clustered to show maximal differentiation along the first and second principal component (PC1 and PC2). Individuals do not cluster according to their urban or rural origin, indicating the absence of population structure.
