# Supplementary material for: Trees and their seed networks: The social dynamics of urban fruit trees and implications for genetic diversity
Source: PLoS One. 2021 Mar 16;16(3):e0243017. doi: 10.1371/journal.pone.0243017 (PMC7963046; doi:10.1371/journal.pone.0243017)
Supplement: S4 Fig — S4a: Changes in K values from the mean log-likelihood probabilities (right axis) and plot of mean likelihood L(K) and variance per K value (left axis) from STRUCTURE runs where inferred clusters (K) ranged from 1 to 10. S4b: Output of clustering analysis by STRUCTURE software for two clusters (K = 2) of the 849 trees, using fourteen microsatellite markers, grouped by origin (urban trees, rural trees). Each vertical bar represents one individual and shows its inferred cluster membership; black and gray colors correspond each to one cluster. If both colors are present, the haplotype consists of a mixture of markers assigned to both black and gray clusters. The samples from the urban area and rural area were assigned in different proportions to each cluster. Using an assignment probability threshold of 0.8, 41% and 8% of individuals from the urban population were respectively assigned to the black and gray clusters (51% were presenting intermediate genotypes) and 5% and 45% of individuals from the rural population were respectively assigned to the black and grey clusters (50% were presenting intermediate genotypes). (PDF) [file pone.0243017.s004.pdf]

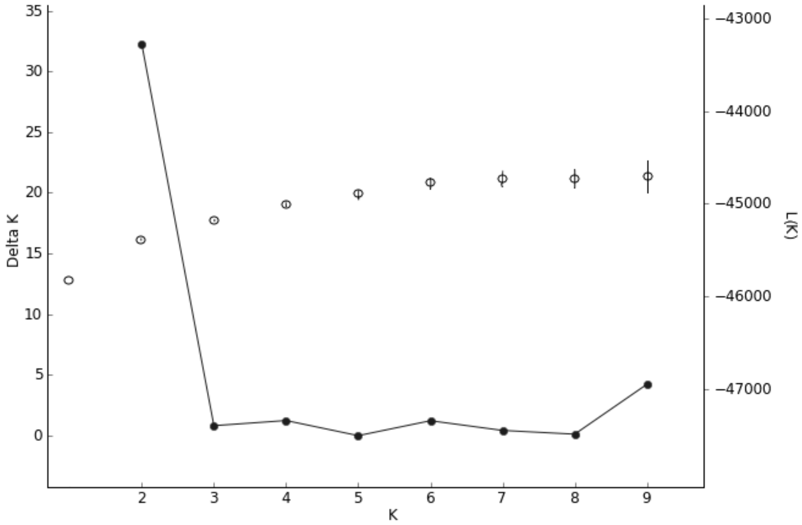

S4a Figure: Changes in K values from the mean log-likelihood probabilities (right axis) and plot of mean likelihood L(K) and variance per K value (left axis) from STRUCTURE runs where inferred clusters (K) ranged from 1 to 10.

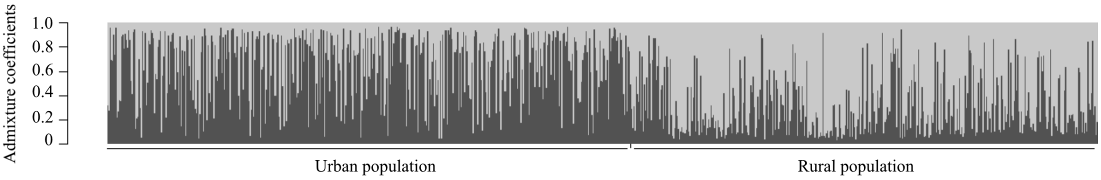

S4b Figure: Output of clustering analysis by STRUCTURE software for two clusters (K=2) of the 849 trees, using fourteen microsatellite markers, grouped by origin (urban trees; rural trees). Each vertical bar represents one individual and shows its inferred cluster membership; black and gray colors correspond each to one cluster. If both colors are present, the haplotype consists of a mixture of markers assigned to both black and gray clusters. The samples from the urban area and rural area were assigned in different proportions to each cluster. Using an assignment probability threshold of 0.8, 41% and 8% of individuals from the urban population were respectively assign to the black and gray clusters (51% were presenting intermediate genotypes) and 5% and 45% of individuals from the rural population were respectively assigned to the black and grey clusters (50% were presenting intermediate genotypes).
