# Supplementary figures and images for: Trees and their seed networks: The social dynamics of urban fruit trees and implications for genetic diversity
Source: PLoS One. 2021 Mar 16;16(3):e0243017. doi: 10.1371/journal.pone.0243017 (PMC7963046; doi:10.1371/journal.pone.0243017)

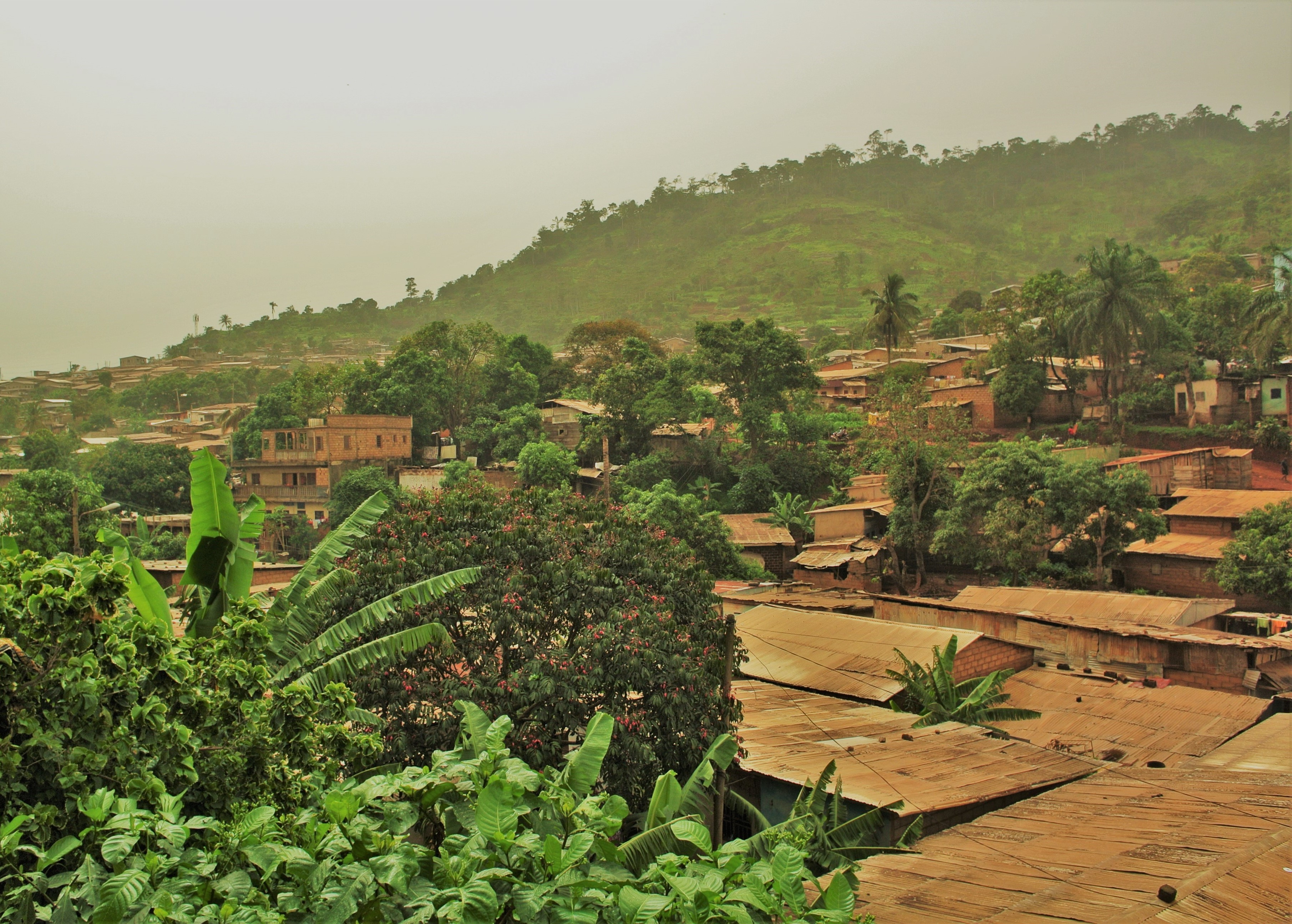

Supplement: S5 Fig — (JPG) [file pone.0243017.s005.jpg]
