# Supplementary material for: Trees and their seed networks: The social dynamics of urban fruit trees and implications for genetic diversity
Source: PLoS One. 2021 Mar 16;16(3):e0243017. doi: 10.1371/journal.pone.0243017 (PMC7963046; doi:10.1371/journal.pone.0243017)
Supplement: Attachment — Submitted filename: PONE_response_to_reviewers.docx [file pone.0243017.s007.docx]

| PONE-D-20-35472

| Trees and their seed networks: the social dynamics of urban fruit trees and implications for genetic diversity

| PLOS ONE

|

Dear authors,

Thank you for submitting your manuscript to PLOS ONE. After careful consideration, we feel that it has merit but does not fully meet PLOS ONE’s publication criteria as it currently stands. Therefore, we invite you to submit a revised version of the manuscript that addresses the points raised during the review process.

Please submit your revised manuscript by 28th, Feb. 2021. If you will need more time than this to complete your revisions, please reply to this message or contact the journal office at plosone@plos.org. When you're ready to submit your revision, log on to https://www.editorialmanager.com/pone/ and select the 'Submissions Needing Revision' folder to locate your manuscript file.

Please include the following items when submitting your revised manuscript:

* A rebuttal letter that responds to each point raised by the academic editor and reviewer(s). You should upload this letter as a separate file labeled 'Response to Reviewers'.

* A marked-up copy of your manuscript that highlights changes made to the original version. You should upload this as a separate file labeled 'Revised Manuscript with Track Changes'.

* An unmarked version of your revised paper without tracked changes. You should upload this as a separate file labeled 'Manuscript'.

If you would like to make changes to your financial disclosure, please include your updated statement in your cover letter. Guidelines for resubmitting your figure files are available below the reviewer comments at the end of this letter.

If applicable, we recommend that you deposit your laboratory protocols in protocols.io to enhance the reproducibility of your results. Protocols.io assigns your protocol its own identifier (DOI) so that it can be cited independently in the future. For instructions see: [ http://journals.plos.org/plosone/s/submission-guidelines#loc-laboratory-protocols | http://journals.plos.org/plosone/s/submission-guidelines#loc-laboratory-protocols ]

We look forward to receiving your revised manuscript.

Kind regards,

Ji-Zhong Wan

Academic Editor

PLOS ONE

Journal Requirements:

When submitting your revision, we need you to address these additional requirements.

1. Please ensure that your manuscript meets PLOS ONE's style requirements, including those for file naming. The PLOS ONE style templates can be found at

https://journals.plos.org/plosone/s/file?id=wjVg/PLOSOne_formatting_sample_main_body.pdf and

https://journals.plos.org/plosone/s/file?id=ba62/PLOSOne_formatting_sample_title_authors_affiliations.pdf

***We checked the style requirements and addressed the different points that were not in compliance with them.

2. We note that Figures 1 and 2 and S2 Figure in your submission contain map and satellite images which may be copyrighted.

All PLOS content is published under the Creative Commons Attribution License (CC BY 4.0), which means that the manuscript, images, and Supporting Information files will be freely available online, and any third party is permitted to access, download, copy, distribute, and use these materials in any way, even commercially, with proper attribution. For these reasons, we cannot publish previously copyrighted maps or satellite images created using proprietary data, such as Google software (Google Maps, Street View, and Earth). For more information, see our copyright guidelines: http://journals.plos.org/plosone/s/licenses-and-copyright.

We require you to either (a) present written permission from the copyright holder to publish these figures specifically under the CC BY 4.0 license, or (b) remove the figures from your submission:

a. You may seek permission from the original copyright holder of Figures 1 and 2 and S2 Figure to publish the content specifically under the CC BY 4.0 license.

We recommend that you contact the original copyright holder with the Content Permission Form ( [ about:blank | http://journals.plos.org/plosone/s/file?id=7c09/content-permission-form.pdf ] ) and the following text:

“I request permission for the open-access journal PLOS ONE to publish XXX under the Creative Commons Attribution License (CCAL) CC BY 4.0 ( [ about:blank | http://creativecommons.org/licenses/by/4.0/ ] ). Please be aware that this license allows unrestricted use and distribution, even commercially, by third parties. Please reply and provide explicit written permission to publish XXX under a CC BY license and complete the attached form.”

Please upload the completed Content Permission Form or other proof of granted permissions as an "Other" file with your submission.

In the figure caption of the copyrighted figure, please include the following text: “Reprinted from [ref] under a CC BY license, with permission from [name of publisher], original copyright [original copyright year].”

b. If you are unable to obtain permission from the original copyright holder to publish these figures under the CC BY 4.0 license or if the copyright holder’s requirements are incompatible with the CC BY 4.0 license, please either i) remove the figure or ii) supply a replacement figure that complies with the CC BY 4.0 license. Please check copyright information on all replacement figures and update the figure caption with source information. If applicable, please specify in the figure caption text when a figure is similar but not identical to the original image and is therefore for illustrative purposes only.

The following resources for replacing copyrighted map figures may be helpful:

USGS National Map Viewer (public domain): [ about:blank | http://viewer.nationalmap.gov/viewer/ ]

The Gateway to Astronaut Photography of Earth (public domain): [ about:blank | http://eol.jsc.nasa.gov/sseop/clickmap/ ]

Maps at the CIA (public domain): [ about:blank | https://www.cia.gov/library/publications/the-world-factbook/index.html ] and [ about:blank | https://www.cia.gov/library/publications/cia-maps-publications/index.html ]

NASA Earth Observatory (public domain): [ about:blank | http://earthobservatory.nasa.gov/ ]

Landsat: http://landsat.visibleearth.nasa.gov/

USGS EROS (Earth Resources Observatory and Science (EROS) Center) (public domain): [ about:blank | http://eros.usgs.gov/# ]

Natural Earth (public domain): [ about:blank | http://www.naturalearthdata.com/ ]

***We replaced the satellite maps with the open ones from Natural Earth (Figure 2 and S2Fig) and from Natural Earth and OpenStreetMaps (Figure 1).

3. Please include a separate caption for each figure in your manuscript.

***We added the captions after the end of each paragraph where a figure was cited for the first time.

Additional Editor Comments:

This is an interesting study. However, the authors have to address all the concerns of the three reviewers before publication.

[Note: HTML markup is below. Please do not edit.]

Reviewers' comments:

Reviewer's Responses to Questions

Comments to the Author

1. Is the manuscript technically sound, and do the data support the conclusions?

The manuscript must describe a technically sound piece of scientific research with data that supports the conclusions. Experiments must have been conducted rigorously, with appropriate controls, replication, and sample sizes. The conclusions must be drawn appropriately based on the data presented.

Reviewer #1: Yes

Reviewer #2: Partly

Reviewer #3: Yes

2. Has the statistical analysis been performed appropriately and rigorously?

Reviewer #1: Yes

Reviewer #2: Yes

Reviewer #3: Yes

3. Have the authors made all data underlying the findings in their manuscript fully available?

The [ http://www.plosone.org/static/policies.action#sharing | PLOS Data policy ] requires authors to make all data underlying the findings described in their manuscript fully available without restriction, with rare exception (please refer to the Data Availability Statement in the manuscript PDF file). The data should be provided as part of the manuscript or its supporting information, or deposited to a public repository. For example, in addition to summary statistics, the data points behind means, medians and variance measures should be available. If there are restrictions on publicly sharing data—e.g. participant privacy or use of data from a third party—those must be specified.

Reviewer #1: No

Reviewer #2: Yes

Reviewer #3: Yes

4. Is the manuscript presented in an intelligible fashion and written in standard English?

PLOS ONE does not copyedit accepted manuscripts, so the language in submitted articles must be clear, correct, and unambiguous. Any typographical or grammatical errors should be corrected at revision, so please note any specific errors here.

Reviewer #1: Yes

Reviewer #2: Yes

Reviewer #3: Yes

5. Review Comments to the Author

Please use the space provided to explain your answers to the questions above. You may also include additional comments for the author, including concerns about dual publication, research ethics, or publication ethics. (Please upload your review as an attachment if it exceeds 20,000 characters)

Reviewer #1: This work is a case study on the impact of human activities on the levels and patterns of genetic diversity of an indigenous fruit tree in Yaoundé, Central Africa. It’s seems interesting to me, and both the experiment design and data analyses of this work are clear, logic and reliable. However, genetic diversities of urban trees are influenced by many factors, such as the economic values, availability of wild species, human activities, culture, seeds sources used for seedlings, et al. So, the implication of this study may be limited and depend on the specific tree species used in studying.

1. There are no figure legends for Fig. 1 and Fig. 2.

*** The figure legends were added, Lines 160-164 for Fig 1. and Lines 210-212 for Fig 2 (all the lines mentioned refer to the Manuscript document).

2. There is no description on the Principal component analysis. How was this analysis done?

*** We added the following description in the Methods section (Lines 182-184): “To visualize the distribution of genotypes of the urban and rural populations, a principal component analysis was performed on the microsatellite dataset using the R package adegenet [49].” The detailed script is stored in the file Scripts.R deposited on the OSF repository.

*[49.	Jombart T. adegenet: a R package for the multivariate analysis of genetic markers. Bioinformatics. 2008;24: 1403–1405. doi:10.1093/bioinformatics/btn129]*

3. Line 202, “the weak but significant genetic differentiation, FST = 0.0057, p < 2.2e-16”. The value of FST is quite small, indicating there is no apparent differentiation between urban and rural populations.

*** The genetic differentiation is very low as indicated by the Fst value, however this value is significantly different from zero as indicated by the statistical test (permutation of individuals among all populations). We have now modified the sentence (Line 218) as follow to make this clearer: “(i) the weak but significant genetic differentiation (*F*_ST_ = 0.0057 and is significantly different from 0 as measured by an exact G test, p < 2.2e-16)”.

4. Line 165 and Line 260, Table 1, use Ne for “effective number of alleles”

*** We replaced as suggested (now at Line 173 and 223).

5. How many microsatellite markers are indeed used? In Line 158, the author said that 12 SSR were used in this study, but in the figure legend of S4b Figure, at Line 9, it said “using 14 microsatellite markers”.

***This is actually 14 SSR, we corrected Line 158 (now Line 166) accordingly.

6. The S4_fig is not clear. Please improve its resolution.

***We did so.

7. In the figure legend of S4b Figure, Line 13-17, the urban and rural populations differed significantly in the proportion of the two genetic clusters. This seems to be incompatible with the PCA result and the estimated population differentiation (FST = 0.0057). The authors should check the result of STRUCTURE analysis and make sure the result of STRUCTURE is reasonable or not.

*** We verified by running again the STRUCTURE analysis with a higher number of iterations (40.000 burn-in and 60.000 MCMC) and replicates (R = 20) and obtained the same result. The two analyses rely on different types of computations/assumptions, so that results obtained are not obligatory incompatible. The Bayesian-clustering method implemented in STRUCTURE was able to attribute only half of individuals to one cluster, which was expected given the very weak genetic structure between the two populations (Fst = 0.0057). The genetic structure obtained through the Bayesian clustering analysis is too low to be observed by the PCA analysis.

Reviewer #2: ‘Trees and their seed networks: the social dynamics of urban fruit trees and implications for genetic diversity’ is an interesting study exploring the genetic diversity present in the African plum tree (Dacryodes edulis) in urban areas. The manuscript is well written, follows a clear logic flow and describes an important aspect of our biodiversity.

My main critical point is around the genetic analysis. As these are not natural populations most Hardy-Weinberg assumptions (used for HE, Ho, Fis, Fst) will be violated, i.e. having a closed population. In many cases when working with wild populations HWE assumptions will be slightly violated but in this case seeds / seedlings are coming from all over the place and the trees analysed will most likely never have formed a natural population.

***Many thanks for outlining this potential problem. We totally agree that cultivated populations can sometimes not be in HWE. This would notably be the case in the presence of vegetative reproduction (eg cultivation of specific cultivars), or because there is a strong selection on the material used for plantation (eg seeds coming from only a limited number of selected individuals). In the case of *Dacryodes edulis*, there is no specific bias on the selection of plantation material used by cultivators. Seeds are coming from many different trees and represent a wide range of genetic diversity of original populations (seeds’ genotypes corresponding to mother trees on which the seed were collected + father trees that have pollinated the female tree given the outcrossing nature of the species). In principle we thus do not expect any specific bias that can affect HWE. This is further confirmed by the use of the F_IS_ statistics that actually allows to test for any specific HWE bias in the observed population. We have estimated F_IS_ values of populations by using a correction for the presence of null alleles (method implemented in INEST software), and F_IS_ value were close to zero, indicating that the population can be considered in HWE.

More specific comments:

Material and Methods, Species description: Could you include information about pollinators and seed dispersers, i.e. is gene flow likely to be predominantly long- or short-distance

***We added the following information (Lines 93-96) and references: “It is pollinated by insects, especially Apoidea [34]. In natural populations, apes, birds and mammals act as seed dispersers, but anthropogenic seed dispersal is more important in cultivated populations, as seeds and fruits are commonly exchanged between relatives, neighbours, and villages [35].”

*[34.	Fohouo F-NT, Messi J, Pauly A. Activité de Meliponula erythra sur les fleurs de* Dacryodes edulis *et son impact sur la fructification. Fruits. 2001;56: 179–188. doi:10.1051/fruits:2001121*

*35.	Degrande A, Tadjo P, Takoutsing B, Asaah E, Tsobeng A, Tchoundjeu Z. Getting trees into farmers’ fields: success of rural nurseries in distributing high quality planting material in Cameroon. Small-scale Forestry. 2013;12: 403–420. doi:10.1007/s11842-012-9220-4]*

Results: You’re describing the genetic diversity as ‘high’ but high compared to what? Natural populations?

***Good point. The level of diversity mentioned here is relative to what is generally found in cultivated plants. We have no data on natural populations yet, so we cannot use this comparison. We have replaced by “presents a relatively high genetic diversity (AR=15-16; Table 2)”.

Results: When you say your STRUCTURE analysis clustered your samples into two ‘weakly’ differentiated clusters, what does that mean? STRUCTURE doesn’t usually give strong or weak clusters.

***“Weakly” refers to the relatively low level of genetic differentiation between the two intra-specific genetic clusters and illustrated by the high number of admixed individuals found (about 50% of individuals).

Results: Table 2: As I understand you collected samples from more than 2 populations but averaged and split into 2 populations (Urban and Rural)? If so, was there a difference in AR between the individual populations?

***In the rural area, we indeed sampled in four large populations. We tested for a difference in AR between these four populations (analysis of variance controlling for the loci effect), but there was none (p-value = 0.7277).

Results: As explained above I don’t think you can do HWE based population genetic analysis for this study as the results will be strongly affected by the violation of the assumptions. You can still look at AR and use the STRUCTURE analysis but not HE, HO, FIS, FST. Unless some of your sampled populations are indeed natural and not planted?

***See our reply above.

Results: Please include on how many individuals AR is based on?

***This was done Line 213, adding the number (k=300).

Results: It would be good to have a bit more information about, i.e. how many alleles did you find in total, did all samples work, were markers tested for things like linkage or null alleles

***We completed the table with the number of alleles. The markers were tested for null alleles (see line 173), and the result was taken into account for the STRUCTURE analysis.

Discussion: It would be good if you could demonstrate that the samples you’ve analysed are indeed genetically diverse. If the species has been analysed genetically in natural populations with the same marker set you could such a comparison. Without that it’s not really possible to say whether diversity is actually high or low in urban areas. It’s possible that it’s high but it might also be significantly lower compared to other areas. If no such studies exist, then you should at least discuss this in the discussion.

***Unfortunately, we do not have any information on wild populations on the species (sampling currently ongoing). The aim of our study was primarily to characterize the social dynamics linked to tree planting of the cultivated species *Dacryodes edulis* in a rural and in an urban population, and secondly its influence on spatial patterns of genetic diversity distribution. For this latter point, we compared the level of genetic diversity between the rural and the urban population. Here, we do not intend to characterize the level of genetic diversity between wild ancestral populations and cultivated populations. We acknowledge that the sentence “However, there are cases in which urban trees commercially grown in nurseries come from a mixture of different wild provenances, and thus show a higher genetic diversity than the wild populations [56]” can bring confusion to the reader, so we have decided discarding this point from the manuscript.

Reviewer #3: Dear Editor and Authors,

The manuscript is well-written with valuable insights on the genetic diversity of an urban African plum tree in Central Africa and their extended network.

From my point of view, findings from this study are of importance significance to be published to scientific community, especially for population diversity researches.

However, figures provided are lack of quality, which I think might affect the manuscript's output.

I recommend minor revision for this manuscript, only after figures are replaced with high-quality images.

***We improved the quality of the images submitted.

6. PLOS authors have the option to publish the peer review history of their article ( [ https://journals.plos.org/plosone/s/editorial-and-peer-review-process#loc-peer-review-history | what does this mean? ] ). If published, this will include your full peer review and any attached files.

If you choose “no”, your identity will remain anonymous but your review may still be made public.

Do you want your identity to be public for this peer review? For information about this choice, including consent withdrawal, please see our [ https://www.plos.org/privacy-policy | Privacy Policy ] Reviewer #1: No

Reviewer #2: No

Reviewer #3: Yes: Dzarifah Zulperi

[NOTE: If reviewer comments were submitted as an attachment file, they will be attached to this email and accessible via the submission site. Please log into your account, locate the manuscript record, and check for the action link "View Attachments". If this link does not appear, there are no attachment files.]

While revising your submission, please upload your figure files to the Preflight Analysis and Conversion Engine (PACE) digital diagnostic tool, [ https://pacev2.apexcovantage.com/ | https://pacev2.apexcovantage.com/ ] . PACE helps ensure that figures meet PLOS requirements. To use PACE, you must first register as a user. Registration is free. Then, login and navigate to the UPLOAD tab, where you will find detailed instructions on how to use the tool. If you encounter any issues or have any questions when using PACE, please email PLOS at [ mailto:figures@plos.org | figures@plos.org ] . Please note that Supporting Information files do not need this step.

In compliance with data protection regulations, you may request that we remove your personal registration details at any time. [ https://www.editorialmanager.com/pone/login.asp?a=r | (Remove my information/details) ] . Please contact the publication office if you have any questions.
